# The Epidemics of the Middle Ages

**Published:** 1835-07-01

**Authors:** 


The Epidemics of the Middle Ages. From the German of *J. F. C. Hecker*, M.D.; translated by *B. G. Babington*, M.D. Octavo, pp. 206. Sherwood and Co. 1835.

German industry, so proverbial for its indefatigability, has collected, in three parts, the accounts of three wide-spread devastations—the "black death" (already noticed in our pages, vol. xix. p. 121)—the "dancing mania"—and the "sweating sickness." Although we cannot help thinking that the time and labour employed in such researches as these might be better expended on other subjects, more directly mixed with our daily pursuits, yet we do not mean to deny all utility to these investigations of the erudite German. We have no doubt that, whatever may have been the nature of the black death, and the sweating sickness, the dancing mania had much to do with mind, and the investigation of this epidemic may probably induce the medical philosopher to admit, that the imagination has a larger share in the production of disease than is generally supposed.

"He has, no doubt, already observed, that joy will affect the circulation, grief the digestion, that anger will heat the frame as perniciously as ardent spirits, and that fear will chill it as certainly as ice; but he may not have carried his observation to the extent of perceiving, that not only single and transient effects, but specific diseases are produced through the agency of mental impressions, and he may therefore still be surprised to find that the dances of St. John and of St. Vitus, as they formerly spread by sympathy from city to city, gave rise to the same deviations from bodily health, in all the individuals whom they attacked; that Tarantism was the same disease, whether medically or morally considered, all over Italy; and that the 'Lycanthropia' of the past, and the 'Leaping Ague' of the present times, have each its respective train of peculiar spmptoms." ix.

The moralist will view the matter according to the bent of his own preconceptions or favourite theories, and will probably come to the conclusion, that the ignorance and superstitions of those dark ages were the main causes that subjected the body to the influence of a morbid or excited imagination. The march of intellect and the spread of knowledge have checked these vagaries in our days—but not annihilated them. They have changed their forms rather than their nature, and have entrenched themselves behind a thousand *little* masks, in the Proteian host of dyspeptic and hypochondriacal affections, instead of coming forward in one grotesque and gigantic monstrosity—like the *dance* now under consideration.

In a very short preface, the author broaches a curious idea—though not altogether new—"that the human race, amidst the creation that surrounds it, moves, in body and soul, *as an individual whole.*" Under this grand conception, we might suppose that *epidemics* are to this whole—this *entity*, or collective organic life—what constitutional diseases, as fevers, are to the individual; while *endemics* will correspond with those topical affections—as hepatitis, pneumonia, &c. that affect certain localities of the human body. The idea, after all, is inferior to the conception of the bard of Twickenham—

"All are but parts of one stupendous whole,
Whose body Nature is, and God the soul."

And, moreover, such an hypothesis would not be of the slightest interest, as it would never bear, in the remotest degree, on any one branch of medical science. We may, therefore, leave this brilliant flight of the imagination to repose tranquilly, among the archives of German dreams and transcendental speculations.

The terrors of the black death had not subsided, and the graves of millions of its victims were scarcely closed, when a strange delusion spread over Germany, and " instead of the divinity of our nature, hurried away both body and soul into the magic circle of hellish superstition." This was a convulsion which agitated the human frame in a most extraordinary manner, exciting the astonishment of philosophers, physicians, divines—indeed of all men, for more than two centuries, since which it has totally disappeared. This was the "CHOREA ST. VITI," so termed on account of the Bacchantic leaps by which it was characterized, and the foaming fury that possessed the performers. It was propagated by the sight of the sufferers, like a demoniacal epidemic, over the whole of Germany, and the neighbouring countries to the north-west. In 1374, assemblages of people were seen at Aix-la-Chapelle, united by one common delusion, and exhibiting in the streets the following degrading spectacle.

" They formed circles hand in hand, and appearing to have lost all control over their senses, continued dancing, regardless of the bystanders, for hours together, in wild delirium, until at length they fell to the ground in a state of exhaustion. They then complained of extreme oppression, and groaned as if in the agonies of death, until they were swathed in cloths bound tightly round their waists, on which they again recovered, and remained free from complaint until the next attack. This practice of swathing was resorted to on account of the tympany which followed these spasmodic ravings, but patients were frequently relieved in a less artificial manner, by thumping and trampling upon the parts affected. While dancing they neither saw nor heard, being insensible to external impressions through the senses, but were haunted by visions, their fancies conjuring up spirits whose names they shrieked out; and some of them afterwards asserted that they felt as if they had been immersed in a stream of blood, which obliged them to leap so high. Others, during the paroxysm, saw the heavens open, and the Saviour enthroned with the Virgin Mary, as indeed the religious notions of the age were strangely and variously reflected in their imaginations.

Where the disease was completely developed, the attack commenced with epileptic convulsions. Those affected fell to the ground senseless, panting and labouring for breath. They foamed at the mouth, and suddenly springing up began their dance amidst strange contortions. Yet the malady doubtless made its appearance very variously, and was modified by temporary or local circumstances, whereof non-medical contemporaries but imperfectly noted the essential particulars, accustomed as they were to confound their observation of natural events with their notions of the world of spirits." 5.

In a few months, this epidemic spread from AIX over the Netherlands, and the heavy-headed, broad-bottomed Belgians danced with all the agility of a Gallic "maitre de danse." These dances manifested a strong antipathy to narrow-toed shoes, red colours, and lachrymation. The complaint was originally confined to the lower classes of society; but the priests took great pains to exorcise the evil spirits, which they believed to have taken possession of these people, lest the devils should get among themselves and the laity. We deem it unnecessary to follow the author from town to town, and from province to province, describing the same mania over and over again, as it happened to be modified by the different manners, customs, habitudes, or localities where it prevailed. We think Dr. Hecker has been needlessly minute and prolix in this respect, and very few medical readers will follow him through all the labyrinths of his descriptions.

*Causes.* So far back as the 14th century, St. John's day was solemnized with all sorts of strange and rude customs,—especially bacchanalian dances, &c. which were observed not only in Germany, but in many countries south of that nation. Dr. H. therefore connects the origin of the dancing mania at Aix, with the festival of St. John, A.D. 1374.

"This is rendered so much the more probable, because some months previously the districts in the neighbourhood of the Rhine and the Maine had met with great disasters. So early as February, both these rivers had overflowed their banks to a great extent; the walls of the town of Cologne, on the side next the Rhine, had fallen down, and a great many villages had been reduced to the utmost distress. To this was added the miserable condition of Western and Southern Germany. Neither law nor edict could suppress the incessant feuds of the Barons, and in Franconia especially, the ancient times of club law appeared to be revived. Security of property there was none; arbitrary will everywhere prevailed; corruption of morals and rude power rarely met with even a feeble opposition; whence it arose that the cruel, but lucrative, persecutions of the Jews, were in many places still practised through the whole of this century, with their wonted ferocity. Thus, throughout the western parts of Germany, and especially in the districts bordering on the Rhine, there was a wretched and oppressed populace; and if we take into consideration, that among their numerous bands many wandered about, whose consciences were tormented with the recollection of the crimes which they had committed during the prevalence of the black plague, we shall comprehend how their despair sought relief in the intoxication of an artificial delirium." 25.

The festival, he thinks, brought the malady to a crisis, though its causes had long before been in operation, in weakening the mind, and disordering the digestive organs.

Our author goes on to trace this dancing mania to periods still more remote, and of which imperfect record may still be traced in the libraries of the curious. It was not, however, till the beginning of the sixteenth century that Paracelsus stripped this mania of its mystic and unhallowed character, as a work of dæmons, and traced it to moral and physical causes. He would not admit that the saints had power, or that God was likely to have the will to torment men by diseases. He therefore divides Chorea St. Viti into three kinds—that which arises from imagination (the original form)—that which springs from sensuality (Chorea Lasciva)—and that which depends on corporeal maladies. The communication of disorders by sympathy is strongly insisted on by this original medical reformer, in his own peculiar language, and with great spirit and knowledge of human nature. It was in Paracelsus's time that the dancing mania began to decline—and ultimately to disappear.

Dr. Hecker next proceeds to give an account of the tarantism, or dancing mania of Italy. The St. Vitus dancers were fortunate in the name of their patron saint, which often saved them from opprobrium, and the charge of being possessed by demons. Other fanatics were not so fortunate, being frequently treated with the most relentless cruelty—instance the burning of witches, &c. &c. The disease called TARANTISM, first broke out in Apulia, and thence diffused itself over Italy, where it prevailed as an epidemic during some centuries. Like St. Vitus's dance, lycanthropy, and witchcraft, it has now disappeared, or lost observation, in consequence of being confined to some few insulated cases that are included under the head of hysteria or monomania. It is hardly necessary to state that tarantism was supposed to result from the bite of the tarantula, a spider formerly common in Italy, but the species of which, is now not recognized—none at least, whose poisonous sting will now produce the disease called tarantism.

" The symptoms which Perotti enumerates as consequent on the bite of the tarantula agree very exactly with those decribed by later writers. Those who were bitten, generally fell into a state of melancholy, and appeared to be stupified, and scarcely in possession of their senses. This condition was, in many cases, united with so great a sensibility to music, that, at the very first tones of their favourite melodies, they sprang up, shouting for joy, and danced on without intermission, until they sunk to the ground exhausted and almost lifeless. In others, the disease did not take this cheerful turn. They wept constantly, and as if pining away with some unsatisfied desire, spent their days in the greatest misery and anxiety. Others, again, in morbid fits of love, cast their longing looks on women, and instances of death are recorded which are said to have occurred under a paroxysm of either laughing or weeping." 66.

We need scarcely observe that the foregoing symptoms could have had nothing to do with the bite of a poisonous animal. The latter would have exhibited a very different train of phenomena. The following reasoning is far more germain to the subject.

" The origin of tarantism itself is referrible, with the utmost probability, to a period between the middle and the end of this century, and is consequently contemporaneous with that of the St. Vitus's dance (1374.). The influence of the Roman Catholic religion, connected as this was in the middle ages, with the pomp of processions, with public exercises of penance, and with innumerable practices which strongly excited the imaginations of its votaries, certainly brought the mind to a very favourable state for the reception of a nervous disorder. Accordingly, so long as the doctrines of Christianity were blended with so much mysticism, these unhallowed disorders prevailed to an important extent, and even in our own days we find them propagated with the greatest facility where the existence of superstition produces the same effect in more limited districts, as it once did among whole nations. But this is not all. Every country in Europe, and Italy, perhaps more than any other, was visited during the middle ages by frightful plagues, which followed each other in such quick succession, that they gave the exhausted people scarcely any time for recovery. The oriental bubo-plague ravaged Italy sixteen times between the years 1119 and 1340. Small-pox and measles were still more destructive than in modern times, and recurred as frequently. St. Anthony's fire was the dread of town and country, and that disgusting disease, the leprosy, which, in consequence of the crusades, spread its insinuating poison in all directions, snatched from the paternal hearth innumerable victims who, banished from human society, pined away in lonely huts, whither they were accompanied only by the pity of the benevolent and their own despair. All these calamities, of which the moderns have scarcely retained any recollection, were heightened to an incredible degree by the Black Death, which spread boundless devastation and misery over Italy. Men's minds were every where morbidly sensitive; and as it happens with individuals whose senses, when they are suffering under anxiety, become more irritable, so that trifles are magnified into objects of great alarm, and slight shocks, which would scarcely affect the spirits when in health, give rise in them to severe diseases, so it was with this whole nation, at all times so alive to emotions, and at that period so sorely pressed with the horrors of death." 74.

We deem it not necessary to follow our indefatigable author through all the windings of this mysterious epidemic—nor to effects of the grand remedy—MUSIC.

"Nothing but the flute or the cithern afforded them relief. At their sounds they awoke as it were by enchantment, opened their eyes, and moving slowly at first, according to the measure of the music, were, as the time quickened, gradually hurried on to the most passionate dance. It was generally observable that country people, who were rude, and ignorant of music, evinced on these occasions an unusual degree of grace, as if they had been well practised in elegant movements of the body; for it is a peculiarity in nervous disorders of this kind, that the organs of motion are in an altered condition, and are completely under the control of the overstrained spirits. Cities and villages alike resounded throughout the summer season with the notes of fifes, clarinets, and Turkish drums; and patients were every where to be met with who looked to dancing as their only remedy." 76.

The prevalence of tarantism having rendered the nervous systems of people inordinately sensitive, the various forms of hysteria became also prevalent, and very often took on the exterior of tarantism itself. Dissimulation lent a powerful hand to the real maladies, and increased the numbers prodigiously. This dancing mania or tarantism prevailed during the whole of the 17th century—and Baglivi, one of the best physicians of that time, made it the subject of a dissertation. He supports his history of the symptoms by the testimony of his father, who was an eye-witness of the epidemic. It declined and fell in the beginning of the 18th century, "when all the links which connected it with the middle ages had long since snapped asunder."

The author goes on to describe the dancing mania of Abyssinia—called the Tigretier, from its prevalence in the Tigrè country. The account is chiefly taken from Pierce, "who is every way worthy of credit," and whose descriptions are equally lively and amusing. We cannot, however, bring ourselves to pursue the subject farther—referring those who are curious to the work itself, where they will also find some interesting notices of later epidemics of this kind, such as the jumpers of Cornwall, the convulsions of certain cotton-factories in Lancashire, of Shetland, Berlin, &c. &c. In an appendix, the translator, among some curious morçeaus, has given us the tarantula dance song, set to music by Athan. Kircher, Rome, 1654. It may prove useful in some of the modern chapels, where strange tongues are learnt by inspiration, and where the "ANTIDOTUM TARANTULÆ," will be an excellent accompaniment.

